# Functional Network-Based Statistics Reveal Abnormal Resting-State Functional Connectivity in Minimal Hepatic Encephalopathy

**DOI:** 10.3389/fneur.2019.00033

**Published:** 2019-01-29

**Authors:** Chuanyin Zhan, Hua-Jun Chen, Yong-Qing Gao, Tian-Xiu Zou

**Affiliations:** Department of Radiology, Fujian Medical University Union Hospital, Fuzhou, China

**Keywords:** minimal hepatic encephalopathy, resting-state functional magnetic resonance imaging, brain functional network, cognitive impairment, network based statistic

## Abstract

**Purpose:** Whole-brain functional network analysis is an emerging methodology for exploring the mechanisms underlying hepatic encephalopathy (HE). This study aimed to identify the brain subnetwork that is significantly altered within the functional connectome in minimal HE (MHE), the earliest stage of HE.

**Materials and Methods:** The study enrolled 19 cirrhotic patients with MHE and 19 controls who underwent the resting-state functional magnetic resonance imaging and cognitive assessment based on the Psychometric Hepatic Encephalopathy Score (PHES). A whole-brain functional connectivity (FC) matrix was calculated for each subject. Then, network-based statistical analyses of the functional connectome were used to perform group comparisons, and correlation analyses were conducted to identify the relationships between FC alterations and cognitive performance.

**Results:** MHE patients showed significant reduction of positive FC within a subnetwork that predominantly involved the regions of the default-mode network, such as the bilateral posterior cingulate gyrus, bilateral medial prefrontal cortex, bilateral hippocampus and parahippocampal gyrus, bilateral angular gyrus, and left lateral temporal cortex. Meanwhile, MHE patients showed significant reduction of negative FC between default-mode network regions (such as the bilateral posterior cingulate gyrus, medial prefrontal cortex, and angular gyrus) and the regions involved in the somatosensory network (i.e., bilateral precentral and postcentral gyri) and the language network (i.e., the bilateral Rolandic operculum). The correlations of FC within the default-mode subnetwork and PHES results were noted.

**Conclusion:** Default-mode network dysfunction may be one of the core issues in the pathophysiology of MHE. Our findings support the notion that HE is a neurological disease related to intrinsic brain network disruption.

## Introduction

Minimal hepatic encephalopathy (MHE) is a frequent cognitive complication of hepatic cirrhosis, diagnosed in up to 80% of patients with cirrhosis (1). It represents the mildest form of hepatic encephalopathy (HE) and is characterized by subtle cognitive impairments, but without the recognizable clinical symptoms of HE (2). In MHE, the cognitive abnormalities primarily involve deficits in speed of information processing, attention, executive control, motor ability, and coordination (3). MHE is associated with impaired quality of life and daily function (e.g., driving capability), as well as a higher risk of progression to overt HE (4–7). Recently, increasing attention has been devoted to uncovering the mechanisms underlying MHE, as understanding these mechanisms would be very helpful for the early diagnosis of MHE and prompt therapy, which can contribute to the improvement of prognosis (1, 7).

Whole-brain functional network analysis is an emerging methodology for the investigation of the pathophysiology of MHE. For example, based on whole-brain functional connectivity (FC) analysis at the region of interest (ROI)-wise level, Zhang et al. (8) demonstrated widespread cortical and subcortical network connectivity alterations in MHE patients, particularly disrupted basal ganglia–thalamocortical FC and abnormal intracortical FC (predominantly in the form of decreased connectivity). In addition, based on whole-brain FC analysis at the voxel-wise level, previous studies outlined the whole-brain FC abnormalities (predominantly in the form of decreased connectivity) in cirrhotic patients with cognitive impairments, which are primarily distributed in the default-mode network (DMN), visual network, attention network, and thalamocortical loop (9, 10). Moreover, nearly all of the FC abnormalities mentioned above were found to be correlated with neuropsychological measurements, which indicates the important role of cortical and subcortical functional disorganization in the mechanisms underlying MHE. While the existing whole-brain functional network studies have helped expand our knowledge of the neural substrates underlying MHE, these studies have only examined whole-brain FC with a single seed region or via pairwise coupling. These approaches may fail to elucidate the role of the connections within the larger network; moreover, they do not provide insight into how MHE is related to the restructuring of functional networks, particularly at the connectome level.

A graph theory-based approach has been successfully used to perform functional connectome analysis by modeling the whole brain as a network, in investigations of organizational changes caused by MHE (11, 12). The graph theory-based analysis demonstrated that the topological organization of the functional whole-brain network in MHE patients tends to be more random, less clustered, and less modular (12, 13). Moreover, the topological properties of functional brain networks altered stepwise with the progression of HE (11, 12). Another nonparametric statistical approach, called “network-based statistics” (NBS) (14), has also been used in the field of connectome analysis. NBS is a method that controls the family-wise error rate during the connectome analysis; it is a mass-univariate testing method based on a network component, rather than an individual link. NBS has been validated as a method for localizing dysfunctional brain connectivity and applied in studies of several neuropsychiatric illnesses, such as Alzheimer's disease (15), Parkinson's disease (16), schizophrenia (17), autism (18), and major depression (19). The existing studies have demonstrated that NBS provides a general framework for the characterization of specific network components, which may be helpful for identifying which subnetworks, if any, exhibit significant functional differences in patients vs. healthy controls. Such an understanding of the FC alteration patterns in patients could aid in elucidating the neuropathological mechanisms underlying MHE. However, few whole-brain NBS studies have been conducted on MHE patients, which limits the further clarification of the network pathophysiology of MHE.

To address this issue, we conducted a comprehensive investigation of the whole-brain functional connectome in cirrhotic patients with MHE in order to identify the FC alteration patterns in MHE. Furthermore, we aimed to demonstrate the association between these changes in FC patterns and cognitive decline in MHE patients.

## Materials and Methods

### Participants

This study was approved by the Research Ethics Committee of Fujian Medical University Union Hospital, China. Written informed consent was obtained from each participant prior to the study. Nineteen cirrhotic patients with MHE and 19 healthy controls were included. The demographic and clinical characteristics of all participants are listed in Table 1. There were no significant differences between the two groups in terms of age, gender, or education level. The Child–Pugh score was used to evaluate functional status of the liver for each patient. The Psychometric Hepatic Encephalopathy Score (PHES) examination—including the digit symbol test, number connection test A, number connection test B, the serial dotting test, and the line tracing test—was used for MHE diagnosis as described previously (9).

**Table 1:** Demographic and clinical characteristics of the subjects.

	**HC subjects (*n* = 19)**	**MHE patients (*n* = 19)**	***P*-value**
Age (year)	50.2 ± 9.3	50.2 ± 9.4	0.90
Gender (Male/Female)	16/3	17/2	0.63 (χ^2^ test)
Education level (year)	8.7 ± 1.8	8.8 ± 1.9	0.30
Etiology of cirrhosis (HBV/alcoholism/ HBV+alcoholism/other)	-	11/4/2/2	-
Child-Pugh stage (A/B/C)	-	2/11/6	-
**PHES TESTS**			
Final PHES (score)	0.5 ± 1.0	−7.8 ± 3.4	<0.001
Number connection test A (seconds)	35.9 ± 7.4	59.4 ± 20.6	<0.001
Number connection test B (seconds)	58.3 ± 14.9	128.3 ± 62.0	<0.001
Serial dotting test (seconds)	42.5 ± 6.1	62.3 ± 14.8	<0.001
Digit symbol test (raw score)	44.8 ± 11.0	28.9 ± 10.0	<0.001
Line tracing test (raw score)	112.7 ± 18.8	192.5 ± 36.3	<0.001

*MHE, minimal hepatic encephalopathy; HC, healthy control; PHES, psychometric hepatic encephalopathy score; HBV, Hepatitis B Virus*.

The exclusion criteria were as follows: (1) diagnosis of current overt HE or another neuropsychiatric disorder; (2) currently taking psychotropic medications; (3) diagnosis of uncontrolled endocrine or metabolic disease (e.g., thyroid dysfunction); (4) alcohol abuse within 6 months prior to the study; (5) contraindications for MRI.

### MRI Data Acquisition

MR Imaging was performed using a 3.0 T scanner (Siemens, Verio, Germany). Three-dimensional T1-weighted magnetization-prepared rapid gradient echo (MPRAGE) sagittal images were collected with the following parameters: TR = 1.9 ms, TE = 2.48 ms, matrix = 256 × 256, FOV = 256 × 256 mm, flip angle = 9°, slice thickness = 1.0 mm (without interslice gap), and 176 slices. Functional images were collected with an echo-planar imaging sequence using the following parameters: TR = 2,000 ms, TE = 25 ms, matrix = 64 × 64, FOV = 240 × 240 mm, flip angle = 90°, slice thickness = 4 mm (without interslice gap), 35 contiguous axial slices, and 180 volumes. Participants were instructed to keep their eyes closed, not think about anything specific, and remain still.

### Functional MRI Data Preprocessing

SPM software and the Data Processing Assistant for Resting-State fMRI tool (DPARSF 3.0, http://www.restfmri.net/forum/DPARSF) were used to preprocess functional MRI data. The first 10 volumes were discarded to reduce the influence of the unstable initial MR imaging signal. Then, slice timing correction and realignment for head motion correction were performed. A subject was excluded if his/her translational movement was >2.0 mm and the rotation was >2.0° (20, 21). The individual T1-weighted images were coregistered to the mean functional images. A unified segmentation algorithm was then used to segment the transformed structural images into gray matter, white matter, and cerebrospinal fluid. Then, based on the normalization parameters estimated during unified segmentation, the motion-corrected functional images were further normalized to the standard Montreal Neurological Institute (MNI) space and resampled to 3 × 3 × 3 mm. Subsequently, the functional images were spatially smoothed using a Gaussian kernel with 4 mm full width at half maximum and were linearly detrended. The resulting data were further bandpass-filtered (0.01–0.08 Hz) to reduce the low-frequency drift and high-frequency physiological respiratory and cardiac noise. To control for the possible effects of head motion and of the global white matter and cerebrospinal fluid signals on the results, we used linear regression to remove several sources of spurious variance, including six head motion parameters and the average signals from the cerebrospinal fluid, white matter, and whole brain.

### Network-Based Statistics

In order to obtain a whole-brain FC matrix for each subject, we defined a set of 90 nodes across the brain based on the Automated Anatomical Labeling (AAL) atlas. For a given node region, the mean time course was calculated as the average of the fMRI time series from all voxels within that region. Then, the correlation matrices could be obtained by computing the Pearson correlation coefficient between the mean time course of each pair of nodes. Thereby, we were able to assess the FC differences between specific pairs of cerebral nodes. The network-based statistics (NBS) tool developed by Zalesky et al. (14) was used for the statistical comparison. Compared to the alternative methods for analyzing resting-state fMRI data (such as seed-based correlation approach and independent components analysis, ICA), NBS consider the whole brain as an integrated system (a connected network) rather than a collection of individual components (22). Thus, NBS allows the higher dimensional analysis rather than the low-dimensional ICA and seed-based correlation analysis, which can make a more comprehensive examination of network connections possible (23). Meanwhile, as a validated statistical method to deal with multiple comparisons during the whole-brain connectivity analysis, NBS can yield substantially greater statistical power than generic procedures, if any connected component (subnetwork) in which the connectivities show significant between-group difference exists (14). In other words, NBS could be more sensitive to identify which subnetwork, if any, exhibit significant functional differences between patients and controls. In this study, to compare the FC matrices of the two groups, a two-sample *t*-test was used with 5,000 permutations, and the significant *P* < 0.05 was corrected for multiple comparisons using NBS correction (14). The significance was measured based on extent, not intensity. Notably, NBS controls for multiple comparisons through cluster-based thresholding, in which connected components of a network are considered a cluster. In this study, the primary cluster-defining threshold (*t* = 3.1) was used to identify a set of suprathreshold edges. Since NBS results are highly dependent on the initial cluster-defining threshold, we performed an additional series of NBS analyses with distinct initial cluster-defining thresholds (*t* = 2.8–3.4) as described in a previous study (24). Very similar results were obtained from these analyses (see Supplementary Figure 1); therefore, we only show the result of NBS analysis with the median cluster-defining threshold (*t* = 3.1) in this article.

### Correlation Analysis

First, the strength of the FC within each subnetwork identified by NBS analysis was calculated. Then, a nonparametric approach, Spearman correlation analysis, was used to assess the association between patients' cognitive performance and FC strength. To correct for multiple comparisons, a false discovery rate (FDR) procedure was performed at *q* < 0.05.

## Results

MHE patients performed significantly worse on all PHES subtests and therefore had lower PHES scores than controls. This result indicates significant cognitive dysfunction in MHE patients.

**Figure 1:**
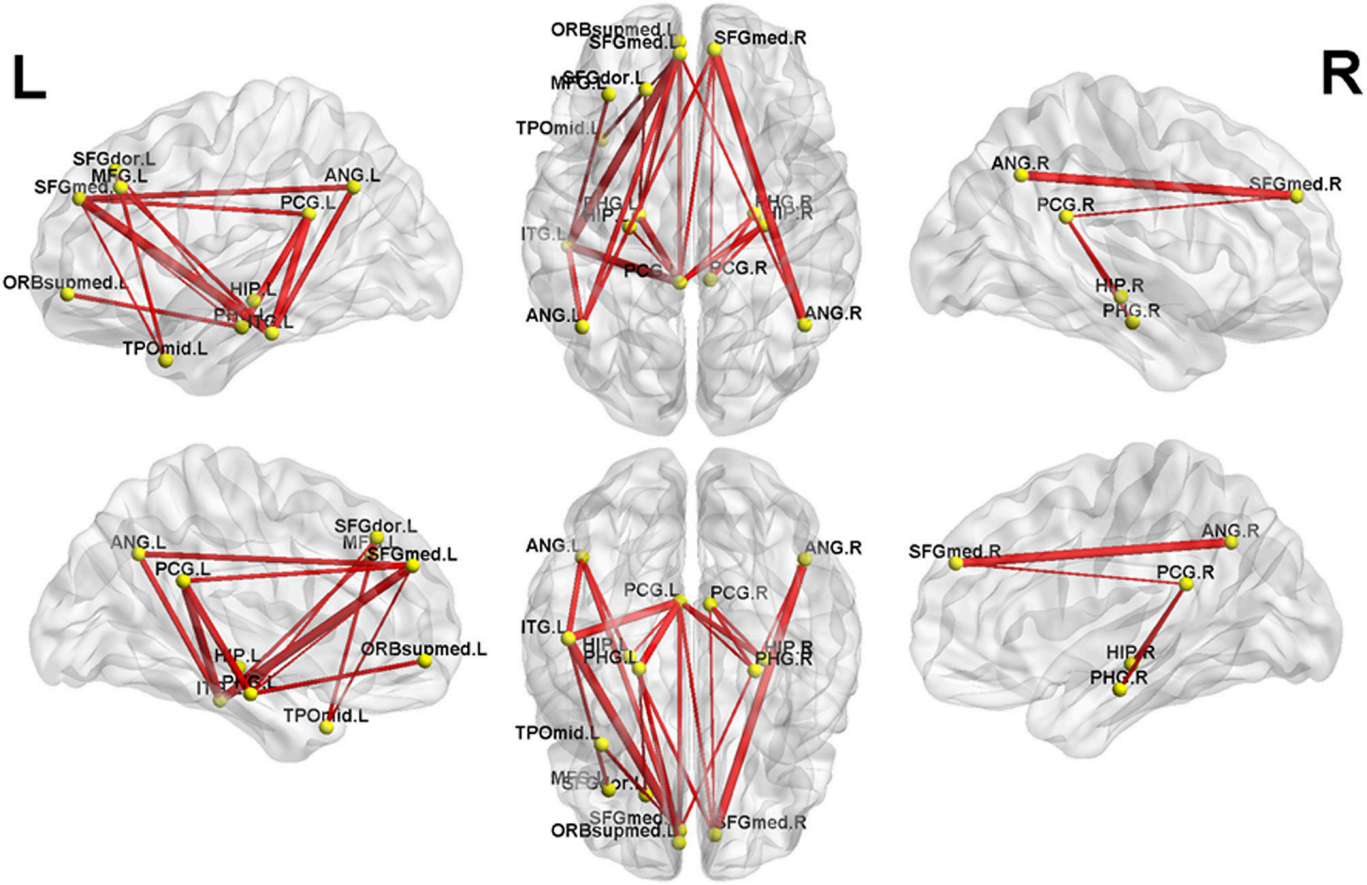
The first subnetwork identified by network-based statistics. This subnetwork predominantly involves the regions of the default-mode network. The red line indicates the positive functional connectivity within this subnetwork. The edge size points out the significance of between-group difference in functional connectivity strength (see Table 2). SFGmed, superior frontal gyrus, medial; PCG, posterior cingulate gyrus; SFGdor, superior frontal gyrus, dorsolateral; HIP, hippocampus; PHG, parahippocampal gyrus; ORBsupmed, superior frontal gyrus, medial orbital; ANG, angular gyrus; TPOmid, temporal pole of middle temporal gyrus; MFG, middle frontal gyrus; ITG, inferior temporal gyrus. The letters “L” and “R” indicate left and right side, respectively.

**Figure 2:**
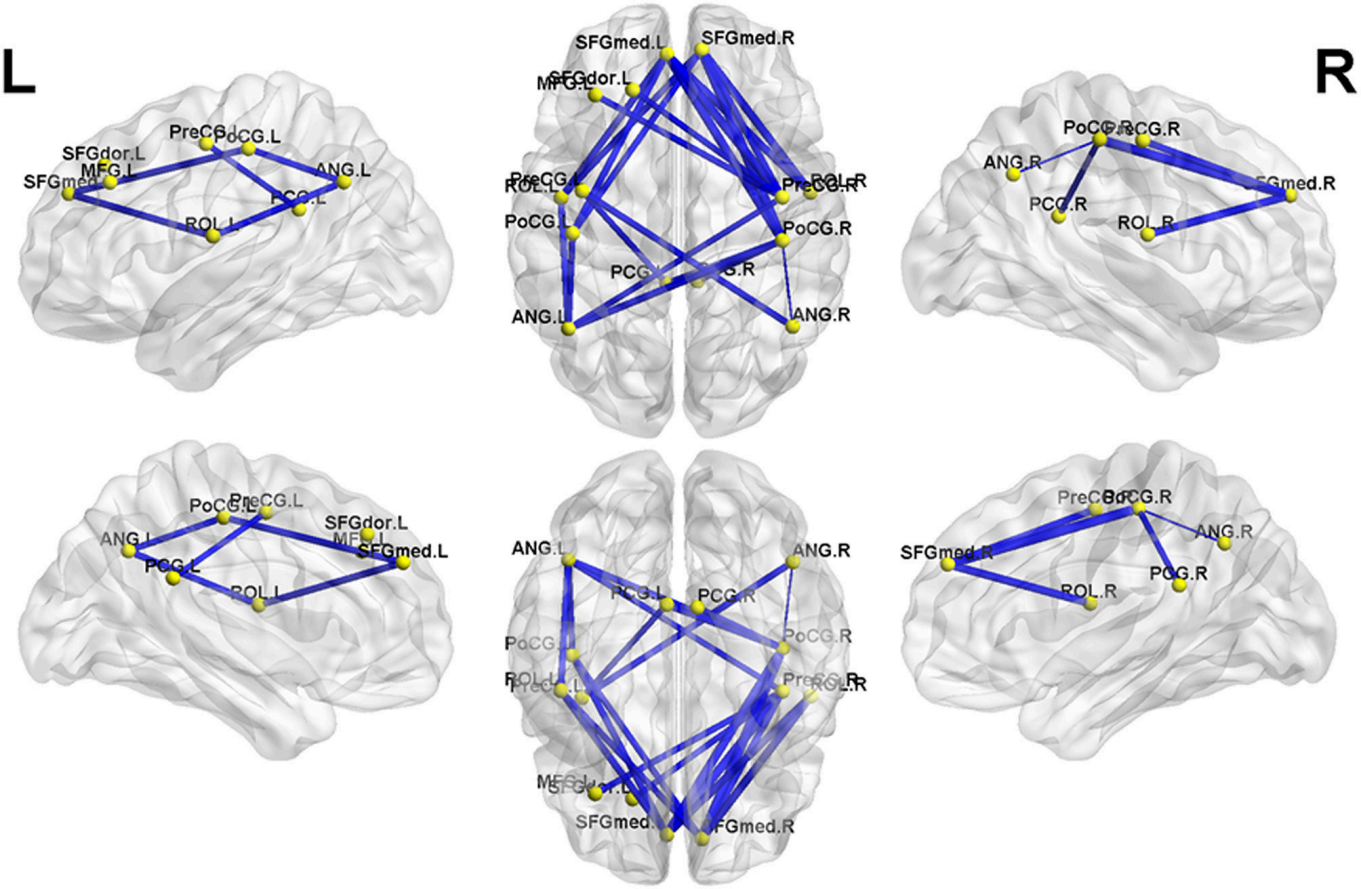
The second subnetwork identified by network-based statistics. This subnetwork mainly involves the regions involved in the default-mode network, somatosensory network, and language network. The blue line indicates the negative functional connectivity within this subnetwork. The edge size points out the significance of between-group difference in functional connectivity strength (see Table 3). PreCG, precental gyrus; SFGdor, superior frontal gyrus, dorsolateral; MFG, middle frontal gyrus; SFGmed, superior frontal gyrus, medial; ROL, Rolandic operculum; PoCG, postcentral gyrus; PCG, posterior cingulate gyrus; ANG, angular gyrus. The letters “L” and “R” indicate left and right side, respectively.

NBS analysis identified two subnetworks with significant differences in FC matrices between patients and controls. The first subnetwork consisted of 15 nodes and 22 edges, with the edges representing a positive functional correlation among the nodes in the controls (Figure 1 and Table 2). The second subnetwork consisted of 14 nodes and 21 edges; in this subnetwork, the edges represented a negative functional correlation among the nodes in the controls (Figure 2 and Table 3).

**Table 2:** Functional connectivity within the first subnetwork identified by NBS analysis.

**Connectivity**	**Functional connectivity strength (Pearson correlation coefficient)**		***T-*value**
	**HC subjects**	**MHE patients**	
SFGmed.L to PCG.L	0.58 ± 0.15	0.38 ± 0.21	3.55
SFGmed.L to PHG.L	0.10 ± 0.17	−0.06 ± 0.14	3.10
SFGmed.L to ANG.L	0.65 ± 0.13	0.41 ± 0.24	3.81
SFGmed.L to ANG.R	0.32 ± 0.20	0.10 ± 0.21	3.24
SFGmed.L to TPOmid.L	0.36 ± 0.17	0.17 ± 0.19	3.19
SFGmed.L to ITG.L	0.27 ± 0.16	−0.01 ± 0.21	4.58
SFGmed.R to PCG.L	0.54 ± 0.19	0.34 ± 0.19	3.34
SFGmed.R to PCG.R	0.46 ± 0.22	0.25 ± 0.19	3.11
SFGmed.R to ANG.L	0.55 ± 0.18	0.29 ± 0.28	3.43
SFGmed.R to ANG.R	0.52 ± 0.22	0.22 ± 0.19	4.53
ORBsupmed.L to PHG.L	0.31 ± 0.18	0.08 ± 0.21	3.55
PCG.L to HIP.L	0.28 ± 0.19	0.04 ± 0.26	3.14
PCG.L to HIP.R	0.24 ± 0.18	−0.01 ± 0.23	3.77
PCG.L to PHG.L	0.25 ± 0.16	0.00 ± 0.21	4.13
PCG.L to PHG.R	0.17 ± 0.14	−0.03 ± 0.22	3.20
PCG.L to ITG.L	0.20 ± 0.20	−0.09 ± 0.21	4.38
PCG.R to HIP.R	0.26 ± 0.19	0.05 ± 0.21	3.34
PCG.R to PHG.R	0.23 ± 0.14	0.03 ± 0.21	3.41
ANG.L to ITG.L	0.39 ± 0.17	0.14 ± 0.23	3.86
SFGdor.L to PHG.L	0.04 ± 0.18	−0.14 ± 0.17	3.22
SFGdor.L to TPOmid.L	0.17 ± 0.17	0.00 ± 0.13	3.46
MFG.L to ITG.L	0.31 ± 0.22	0.06 ± 0.23	3.45

*SFGmed, superior frontal gyrus, medial; PCG, posterior cingulate gyrus; SFGdor, superior frontal gyrus, dorsolateral; HIP, hippocampus; PHG, parahippocampal gyrus; ORBsupmed, superior frontal gyrus, medial orbital; ANG, angular gyrus; TPOmid, temporal pole: middle temporal gyrus; MFG, middle frontal gyrus; ITG, inferior temporal gyrus. The letters “L” and “R” indicate left and right side, respectively*.

**Table 3:** Functional connectivity within the second subnetwork identified by NBS analysis.

**Connectivity**	**Functional connectivity strength (Pearson correlation coefficient)**		***T-*value**
	**HC subjects**	**MHE patients**	
SFGmed.L to PreCG.R	−0.43 ± 0.16	−0.15 ± 0.20	4.68
SFGmed.L to PoCG.L	−0.39 ± 0.18	−0.13 ± 0.26	3.50
SFGmed.L to PoCG.R	−0.48 ± 0.18	−0.15 ± 0.24	4.66
SFGmed.L to ROL.L	−0.44 ± 0.18	−0.18 ± 0.26	3.60
SFGmed.L to ROL.R	−0.47 ± 0.18	−0.18 ± 0.27	4.01
SFGmed.R to PreCG.R	−0.42 ± 0.20	−0.18 ± 0.22	3.57
SFGmed.R to PoCG.L	−0.40 ± 0.19	−0.17 ± 0.23	3.24
SFGmed.R to PoCG.R	−0.50 ± 0.17	−0.16 ± 0.23	5.06
SFGmed.R to ROL.L	−0.44 ± 0.20	−0.22 ± 0.22	3.33
SFGmed.R to ROL.R	−0.46 ± 0.18	−0.19 ± 0.21	4.20
PCG.L to PreCG.L	−0.30 ± 0.18	−0.10 ± 0.23	3.11
PCG.L to PoCG.R	−0.35 ± 0.18	−0.13 ± 0.20	3.65
PCG.R to PoCG.R	−0.34 ± 0.23	−0.13 ± 0.18	3.14
ANG.L to PreCG.R	−0.44 ± 0.17	−0.23 ± 0.24	3.10
ANG.L to PoCG.L	−0.40 ± 0.18	−0.11 ± 0.34	3.28
ANG.L to PoCG.R	−0.47 ± 0.17	−0.22 ± 0.24	3.68
ANG.L to ROL.L	−0.46 ± 0.19	−0.19 ± 0.29	3.48
ANG.R to PreCG.L	−0.34 ± 0.20	−0.11 ± 0.22	3.45
ANG.R to PoCG.R	−0.48 ± 0.15	−0.26 ± 0.21	3.53
SFGdor.L to PreCG.R	−0.40 ± 0.17	−0.19 ± 0.25	3.12
MFG.L to PreCG.R	−0.35 ± 0.15	−0.13 ± 0.25	3.35

*PreCG, precentral gyrus; SFGdor, superior frontal gyrus, dorsolateral; MFG, middle frontal gyrus; SFGmed, superior frontal gyrus, medial; ROL, Rolandic operculum; PoCG, postcentral gyrus; PCG, posterior cingulate gyrus; ANG, angular gyrus. The letters “L” and “R” indicate left and right side, respectively*.

Compared to controls, MHE patients showed a significant reduction of positive FC within the first subnetwork (Figure 1 and Table 2). This subnetwork predominantly involved the regions of the DMN, such as the bilateral posterior cingulate gyrus, bilateral medial prefrontal cortex, bilateral hippocampus and parahippocampal gyrus, bilateral angular gyrus, and left lateral temporal cortex (left middle temporal gyrus [temporal pole part] and left inferior temporal gyrus). Additionally, this subnetwork featured connectivity of the left superior frontal gyrus (dorsolateral part) with the left parahippocampal gyrus and connectivity of the left superior frontal gyrus (dorsolateral part) with the left middle temporal gyrus (temporal pole part). Connectivity between the left middle frontal gyrus and left inferior temporal gyrus was also found in this subnetwork.

Meanwhile, in the second subnetwork, MHE patients showed a significant reduction of negative FC (Figure 2 and Table 3). This subnetwork predominantly involved connectivities between DMN regions (such as the bilateral posterior cingulate gyrus, medial prefrontal cortex, and angular gyrus) and the bilateral precentral and postcentral gyrus and bilateral Rolandic operculum. Additionally, connectivity of the right precentral gyrus with the left superior frontal gyrus (dorsolateral part) and connectivity of the right precentral gyrus with the left middle frontal gyrus were also found in this subnetwork.

In the patient group, Spearman correlation analysis revealed that the PHES score was significantly positively correlated with the strength of two FCs within the first subnetwork, namely, the connectivity between the left posterior cingulate gyrus and right hippocampus (*r* = 0.556, *P* = 0.044, FDR-corrected) and the connectivity between the left superior frontal gyrus (dorsolateral part) and left parahippocampal gyrus (*r* = 0.477, *P* = 0.039, uncorrected).

## Discussion

In the present study, we performed NBS analysis to identify FC changes related to MHE at the whole-brain functional connectome level for the first time. Network-wise FC changes were found in two brain subnetworks with significant FC reduction: One predominantly involved positive FC among DMN regions, including the bilateral posterior cingulate gyrus, bilateral medial prefrontal cortex, bilateral hippocampus and parahippocampal gyrus, bilateral angular gyrus, and left lateral temporal cortex; the other primarily involved negative FC among DMN regions (such as the bilateral posterior cingulate gyrus, medial prefrontal cortex, and angular gyrus) and regions that engage in the somatosensory network (i.e., bilateral precentral and postcentral gyri) and language network (i.e., bilateral Rolandic operculum). We also identified the correlations between FC within the default-mode subnetwork and MHE patients' cognitive performance. These findings provide new insights into the mechanisms of MHE.

The reduction in both positive and negative FC revealed by our study was consistent with the findings of previous whole-brain functional network analyses. In studies based on a seed region or conducted via pairwise coupling, MHE patients have always shown widespread FC abnormalities (predominantly decreased FC), especially in the DMN, attention network, and thalamocortical circuit (8–10, 25). Additionally, whole-brain functional connectome analyses using the graph theory-based method have revealed significant reductions of connectivity strength in MHE, which are predominantly located in bilateral midline brain areas and bilateral primary somatosensory and auditory regions (12). Notably, previous whole-brain FC analyses also found FC increases related to MHE in several regions involving the somatosensory network, visual network, and memory system, which is regarded as a compensatory mechanism (8, 9). We did not find such compensatory FC changes in the present study. There are two likely reasons for this discrepancy: differences in the samples (e.g., patients with heterogeneous etiologies of cirrhosis) and different data analysis methods with distinct sensitivity and pertinence.

The first brain subnetwork identified by NBS analysis in our study mainly included the DMN regions. This pattern is consistent with previous reports in which both static and dynamic resting-state FC showed disruption in the DMN (particularly in the medial prefrontal cortex and posterior cingulate gyrus) of patients with cirrhosis and MHE (26–29). These default-mode connectivity changes may be attributed to brain regional edema (30, 31) and impairment of the microstructural integrity of white matter fiber (31, 32). Furthermore, previous MHE studies also demonstrated a significant association between the altered connectivity strength within the DMN and clinical parameter (e.g., blood ammonia level and neuropsychological performance) abnormalities as well as the development and prognosis of HE disease (9, 26, 29, 31). In fact, it has been well-documented that DMN is involved in various cognitive processes, such as memory, attention, and executive functions (33–35). A study on MHE therapy demonstrated that the cognitive amelioration following treatment with oral L-ornithine is correlated with decreases in activation (i.e., increases in deactivation) in the posterior cingulate and ventral medial prefrontal cortex, two pivotal regions of the DMN (36). Thus, the correlations of the FC within the default-mode subnetwork (i.e., the connectivity between the left posterior cingulate gyrus and right hippocampus) and PHES results found this study were not surprising.

The second brain subnetwork identified by NBS analysis predominantly included the connectivity between DMN regions (such as the bilateral posterior cingulate gyrus, medial prefrontal cortex, and angular gyrus) and the bilateral precentral and postcentral gyri and bilateral Rolandic operculum. The bilateral precentral and postcentral gyri represent the primary somatosensory areas, in which the spontaneous neural activity and the energy metabolism are disturbed in MHE patients (37–39). Meanwhile, the bilateral Rolandic operculum is one of the major regions involved in the language processing system, which is especially associated with speech (40). Previous studies have revealed that MHE patients have decreased regional connectivity strength and efficiency in a few brain areas, including the bilateral Rolandic operculum (11, 12). Taken together, these existing reports suggest that MHE could induce dysfunction of the somatosensory and language systems, which is in line with our findings. In fact, neurophysiological and neuropsychological studies have demonstrated deficits in fine motor skills (such as extrapyramidal and cerebellar symptoms) in MHE patients (41, 42). Additionally, although language function is relatively spared in patients with cirrhosis and MHE, there have been some reports of language function decline in MHE (43). Of note, it has been well-documented that the proper FC pattern among the intrinsic brain networks is the key characteristic of human brain functional organization, which plays an important role in functional integration and is essential for various neural functions (44–46). Therefore, our finding that the negative connectivity between DMN regions and the areas of the somatosensory and language networks was decreased in MHE was in line with expectations. The neuropathology mechanisms underlying the FC reduction among these networks in MHE is not well understood and should be further addressed in the future.

We acknowledge several limitations of this work. First, this study had a relatively small sample size. Second, our design did not enable us to control for the potential confounding effects of heterogeneous etiologies of cirrhosis (e.g., the residual effect of past alcohol abuse). Previous studies have demonstrated that distinct etiologies could induce different degrees of brain structural and functional changes in cirrhotic patients (47, 48). Third, we could not evaluate whether network-wise FCs are related to deficits in specific cognitive domains (e.g., memory, attention, and executive domains), since only a general cognition assessment (i.e., the PHES examination) was performed. More comprehensive evaluation specific to the distinct cognitive domains is recommended for future studies. Fourth, the methodological limitations of NBS should be considered. NBS controls for multiple comparisons through cluster-based thresholding, but a widely acknowledged drawback of NBS is the arbitrary nature of threshold choice (14). To address this issue, we repeatedly performed NBS with a range of initial cluster-defining thresholds. The similarly of the results for the various thresholds validated the stability and reliability of our findings.

In summary, our findings indicate that DMN dysfunction may be one of the core issues in the pathophysiology of MHE. The decreased connectivity within the DMN and between the DMN and several networks, such as somatosensory and language networks, is the consistent and key characteristic of early HE and may be responsible for the mechanisms underlying MHE. Our results provide further support for the notion that HE is a neurological disease related to intrinsic brain network disruption.

## Author Contributions

H-JC and T-XZ conceived and designed the study, acquired and analyzed the data, and wrote the manuscript. CZ and Y-QG contributed to data analysis. All authors have read and approved the manuscript.

### Conflict of Interest Statement

The authors declare that the research was conducted in the absence of any commercial or financial relationships that could be construed as a potential conflict of interest.

## Supplementary Material

Supplementary Figure 1 The functional connectivity pattern of the subnetwork identified by network-based statistics with different initial cluster-defining thresholds (*t* = 2.8–3.4). Very similar results were obtained across these analyses.

## Abbreviations

MHE: minimal hepatic encephalopathy
HE: hepatic encephalopathy
DMN: default-mode network
NBS: Network based statistic
PHES: Psychometric Hepatic Encephalopathy Score
FC: functional connectivity.
