# Supplementary figures and images for: Functional Network-Based Statistics Reveal Abnormal Resting-State Functional Connectivity in Minimal Hepatic Encephalopathy
Source: Front Neurol. 2019 Jan 29;10:33. doi: 10.3389/fneur.2019.00033 (PMC6362410; doi:10.3389/fneur.2019.00033)

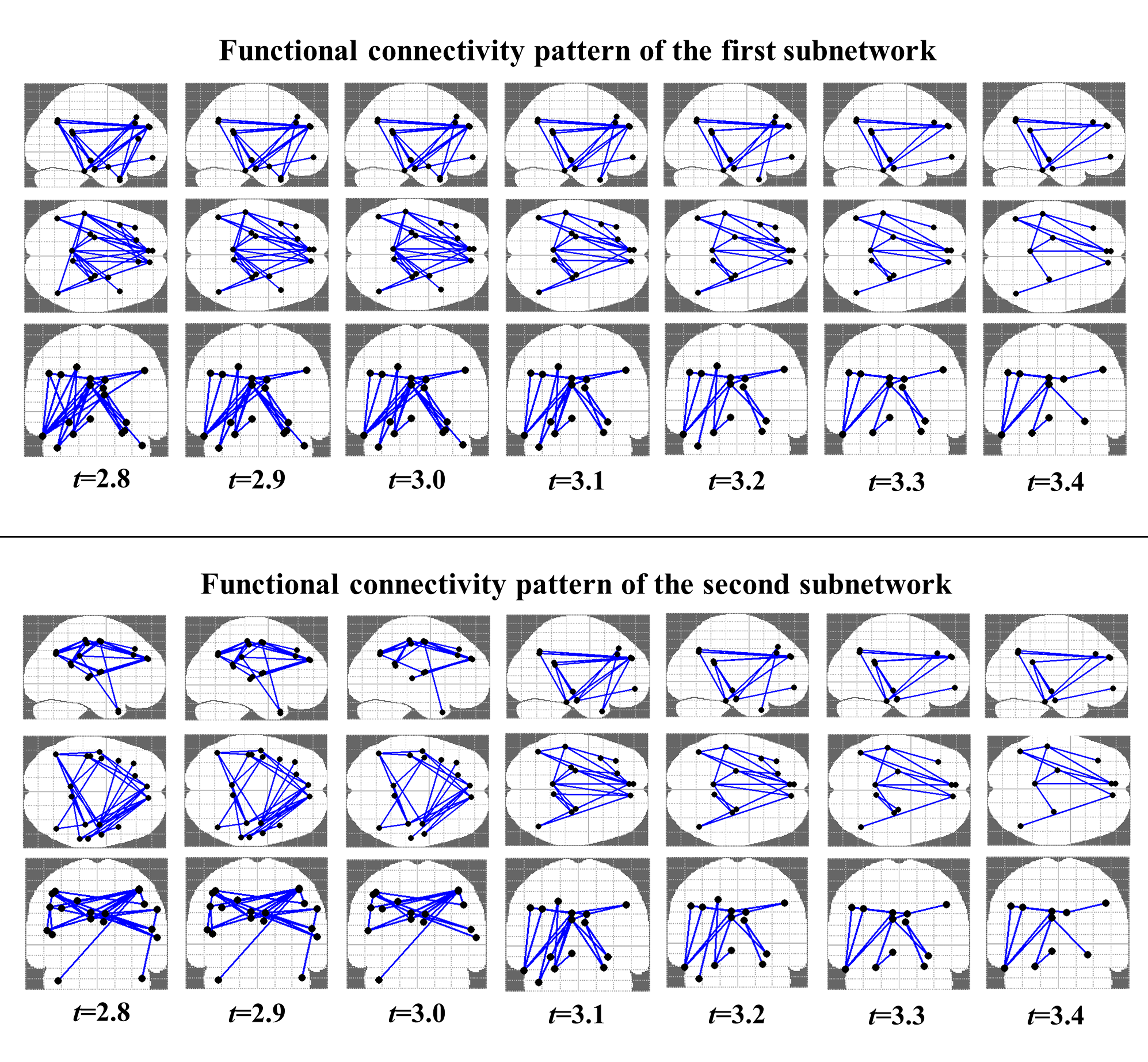

Supplement: Supplementary Figure 1 — The functional connectivity pattern of the subnetwork identified by network-based statistics with different initial cluster-defining thresholds (t = 2.8–3.4). Very similar results were obtained across these analyses. [file Image_1.TIF]
